# Focal adhesion kinase is required for synovial fibroblast invasion, but not murine inflammatory arthritis

**DOI:** 10.1186/s13075-014-0464-6

**Published:** 2014-10-04

**Authors:** Miriam A Shelef, David A Bennin, Nihad Yasmin, Thomas F Warner, Thomas Ludwig, Hilary E Beggs, Anna Huttenlocher

**Affiliations:** Division of Rheumatology, Department of Medicine, University of Wisconsin–Madison and William S Middleton Memorial VA Medical Center, 1685 Highland Ave, Madison, WI 53705 USA; Departments of Pediatrics and Medical Microbiology and Immunology, University of Wisconsin – Madison, 1550 Linden Drive, Madison, WI 53706 USA; Department of Internal Medicine, Aurora Healthcare, 945 N 12th street, Milwaukee, WI 53233 USA; Department of Pathology and Laboratory Medicine, University of Wisconsin – Madison, 600 Highland Ave, Madison, WI 53792 USA; Department of Molecular and Cellular Biochemistry, The Ohio State University, 460 West 12th Avenue, Columbus, OH 43210 USA; Department of Ophthalmology, University of California, 10 Koret Way, San Francisco, CA 94143 USA

## Abstract

**Introduction:**

Synovial fibroblasts invade cartilage and bone, leading to joint destruction in rheumatoid arthritis. However, the mechanisms that regulate synovial fibroblast invasion are not well understood. Focal adhesion kinase (FAK) has been implicated in cellular invasion in several cell types, and FAK inhibitors are in clinical trials for cancer treatment. Little is known about the role of FAK in inflammatory arthritis, but, given its expression in synovial tissue, its known role in invasion in other cells and the potential clinical availability of FAK inhibitors, it is important to determine if FAK contributes to synovial fibroblast invasion and inflammatory arthritis.

**Methods:**

After treatment with FAK inhibitors, invasiveness of human rheumatoid synovial fibroblasts was determined with Matrigel invasion chambers. Migration and focal matrix degradation, two components of cellular invasion, were assessed in FAK-inhibited rheumatoid synovial fibroblasts by transwell assay and microscopic examination of fluorescent gelatin degradation, respectively. Using mice with tumor necrosis factor α (TNFα)–induced arthritis in which fak could be inducibly deleted, invasion and migration by FAK-deficient murine arthritic synovial fibroblasts were determined as described above and arthritis was clinically and pathologically scored in FAK-deficient mice.

**Results:**

Inhibition of FAK in human rheumatoid synovial fibroblasts impaired cellular invasion and migration. Focal matrix degradation occurred both centrally and at focal adhesions, the latter being a novel site for matrix degradation in synovial fibroblasts, but degradation was unaltered with FAK inhibitors. Loss of FAK reduced invasion in murine arthritic synovial fibroblasts, but not migration or TNFα-induced arthritis severity and joint erosions.

**Conclusions:**

FAK inhibitors reduce synovial fibroblast invasion and migration, but synovial fibroblast migration and TNFα-induced arthritis do not rely on FAK itself. Thus, inhibition of FAK alone is unlikely to be sufficient to treat inflammatory arthritis, but current drugs that inhibit FAK may inhibit multiple factors, which could increase their efficacy in rheumatoid arthritis.

## Introduction

Synovial fibroblasts are critical for the pathogenesis of rheumatoid arthritis. These cells normally line the joint, but in rheumatoid arthritis they increase in number as part of the pannus, a tumorlike structure that causes significant joint destruction [1]. Synovial fibroblasts secrete inflammatory cytokines, degrade cartilage and bone [2,3] and can migrate to invade distant cartilage in mouse models [4]. Despite the fact that their ability to invade can be pathologic, little is known about what mediates synovial fibroblast invasion.

Cellular invasion is a multistep process that involves cell adhesion at the site of invasion, formation of invasive structures, focal matrix degradation and migration through the newly degraded area to continue the invasion process. Different cell types generate different structures to invade. Arthritic rat [5] and possibly human rheumatoid [6] synovial fibroblasts make invadopodia, structures often used by cancer cells to invade and metastasize [7]. Cancer cells recently have been shown to also degrade matrix at focal adhesions [8], structures that function primarily as cellular anchors.

Focal adhesion kinase (FAK) is a nonreceptor protein tyrosine kinase and scaffolding protein that mediates numerous cellular functions, including adhesion, migration and invasion [9]. FAK can be found in different parts of the cell, but is often localized to focal adhesions in part through interactions with paxillin [10]. Downstream of integrin binding, FAK becomes activated, which involves autophosphorylation of tyrosine 397 and leads to a signaling cascade ultimately resulting in cytoskeletal reorganization and other activities [11,12]. FAK has been implicated in invasion in normal cells such as macrophages [13], as well as in tumor cells [9]. Further, FAK inhibitors are being studied in clinical trials for cancer treatment [14]. One of these agents, PF-562,271, reduces pancreatic and prostate cancer metastases in mice [15,16], supporting a role for FAK in cellular invasion and metastatic disease *in vivo*.

Given the importance of synovial fibroblast invasion in rheumatoid arthritis, FAK may be important in rheumatoid arthritis pathogenesis. Rheumatoid synovial tissue has increased phosphorylated FAK and tumor necrosis factor α (TNFα) can induce FAK phosphorylation in rheumatoid synovial fibroblasts [17], which induces matrix metalloproteases [9]. Further, rapamycin, which decreases the invasiveness of arthritic synovial fibroblasts, reduces FAK phosphorylation [18]. Pathogen-associated molecular patterns (PAMPs) induce interleukin 6 production in rheumatoid synovial fibroblasts, which requires FAK phosphorylation at tyrosine 397 [19,20]. Apart from a role in synovial fibroblast invasion, FAK has been hypothesized to be important in inflammatory arthritis related to synovial angiogenesis [21] and could also contribute through its role in regulating macrophage invasion [22], neutrophil survival [23], T-cell activation [24] and osteoclast function [25]. PF-573,228, another FAK inhibitor, reduces macrophage recruitment and has antiangiogenic properties [26], and both PF-562,271 and PF-573,228 reduce injury-mediated fibrosis [27,28]. Thus, there are many reasons to suspect that FAK might be important in rheumatoid arthritis and that FAK inhibitors might be beneficial, but little has been done to test the role of FAK in inflammatory arthritis.

In this study, we show that FAK inhibitors reduce rheumatoid synovial fibroblast invasion and migration, but that FAK itself is required only for synovial fibroblast invasion, not migration or murine TNFα-induced arthritis.

## Methods

### Patients

Deidentified synovial fluid samples obtained during the routine care of patients given a diagnosis of rheumatoid arthritis by a rheumatologist were provided by physicians not associated with this research. This research does not meet the definition of human subjects research as confirmed by the University of Wisconsin Institutional Review Board.

### Animals

Mice which overexpress one copy of the TNFα transgene (line 3647) [29] on a C57BL/6 background (from Dr. George Kollias and the Alexander Fleming Biomedical Sciences Research Center, Varkiza, Greece) were crossed with FAK^f/f^ mice on a 129 background [30] and Rosa26-CreER^T2^ mice [31] on a C57BL/6 background to ultimately generate TNF^+^CreER^+^FAK^f/f^ and TNF^+^CreER^−^FAK^f/f^ mice for experiments. Mice were cared for and killed in a manner approved by the University of Wisconsin Animal Care and Use Committee.

### Synovial fibroblast cultures

Synovial fibroblast cultures were prepared from mouse ankle joints and from human synovial fluid as previously described [32,33]. Briefly, murine ankles were minced, incubated with collagenase type IV (Worthington Biochemical, Lakewood, NJ, USA) at 37°C, and large debris was filtered out with a cell strainer. Cells were plated in a culture medium composed of Dulbecco’s modified Eagle’s medium with 10% fetal bovine serum (FBS), L-glutamine, nonessential amino acids, essential amino acids, penicillin, streptomycin and β-mercaptoethanol. Human synovial fluid was centrifuged, and the cell pellet was resuspended in the above media. Synovial fibroblasts were used between passages 5 and 9 unless otherwise stated. Before use, mouse synovial fibroblasts were confirmed to express vascular cell adhesion molecule 1 and not F4/80 or CD45, and human synovial fibroblasts were confirmed to express CD90 and not CD68 (data not shown). Cells were serum-starved overnight before all invasion, migration and matrix degradation experiments were carried out in media identical to that described above, but with only 0.1% FBS.

### Inhibition and depletion of focal adhesion kinase *in vitro*

Human rheumatoid synovial fibroblasts were treated with 5 μM PF-562,271 (Symansis, Timaru, New Zealand), 5 μM PF-573,228 (Tocris Bioscience, Bristol, UK) or an equal volume of dimethyl sulfoxide (DMSO) for 10 minutes at room temperature before and also during migration, invasion or degradation assays. The final concentration of DMSO was 0.5% or less for all experiments. Dosing of drugs and DMSO was based on previous studies [24] and confirmed not to alter cell metabolism, viability and function (data not shown). TNF^+^CreER^+^FAK^f/f^ murine synovial fibroblasts were treated with 1 μM (Z)-4-OH tamoxifen (Sigma-Aldrich, St Louis, MO, USA) in 95% ethanol or vehicle control (95% ethanol) for 4 days before experiments.

### Western blotting

Synovial fibroblasts were scraped into lysis buffer (5 mM Tris, pH 7.4; 500 mM NaCl; 0.1% sodium monododecyl sulfate; 0.5% deoxycholic acid; 0.5 mM MgCl_2_) containing 2 μg/ml aprotinin, 1 μg/ml leupeptin, 1 μg/ml pepstatin, 0.2 mM phenylmethylsulfonyl fluoride and 1 mM sodium vanadate on ice. Spleens were dissociated, and splenocytes were lysed in lysis buffer after red blood cell lysis. Equivalent amounts of cell lysates were subjected to SDS-PAGE and transferred onto nitrocellulose membranes for Western blotting using standard methods. Blots were incubated with dilutions of 1:500 anti-FAK (clone 77; BD Transduction Laboratories, San Jose, CA, USA), 1:100 anti-phospho-Tyr-397 FAK (polyclonal antibody; Invitrogen, Carlsbad, CA, USA), 1:1,000 rabbit anti-tubulin (Cell Signaling Technology, Danvers, MA, USA) and/or 1:1,000 mouse anti-β-actin (clone AC-15; Sigma-Aldrich). Secondary antibodies were used at 1:10,000 and were conjugated to IREDye 680 or 800 (LI-COR Biosciences, Lincoln, NE, USA). Blots were imaged and bands were quantified by densitometry using the Odyssey infrared imaging system (LI-COR Biosciences).

### Invasion assay

Murine arthritic (2.5 ×10^4^) or human rheumatoid (1.5 × 10^4^) synovial fibroblasts were pipetted into the upper wells of Matrigel BioCoat Invasion Chambers (BD Biosciences, San Jose, CA, USA) in 0.1% FBS media. The lower chambers contained 10% FBS media as an attractant. Cells were incubated for 24 hours at 37°C with 10% CO_2_. Noninvaded cells were removed with a cotton swab, and the membranes were fixed in methanol and stained with Hema 3 Manual Staining System (Fisher Scientific, Asheville, NC, USA) according to the manufacturer’s instructions. For each transwell membrane, the number of cells that invaded across the membrane for ten microscopic fields at 100× magnification were counted for human studies and four microscopic fields at 100× magnification for mouse studies.

### Migration assay

Rheumatoid (3 × 10^4^) and murine arthritic (2 × 10^4^) synovial fibroblasts in 0.1% FBS media were placed in the upper chamber of a transwell previously coated with 10 μg/ml fibronectin and blocked with 2% bovine serum albumin in phosphate-buffered saline (PBS). The lower chamber contained 10% FBS media as an attractant. After incubation for 4 hours (murine synovial fibroblasts) or 5 hours (human rheumatoid synovial fibroblasts) at 37°C with 10% CO_2_, nonmigrated cells were removed with a cotton swab and the membranes were fixed, stained and counted in the same manner as was done for invasion assays.

### Gelatin degradation assay

Gelatin coverslips were prepared as published previously [33]. Briefly coverslips were with treated with 50 μg/ml poly-L-lysine, then 0.5% glutaraldehyde, then 0.2% Oregon green gelatin (Molecular Probes, Eugene, OR, USA), then 5 mg/ml NaBH_4_, with PBS washes done between each treatment. Rheumatoid synovial fibroblasts (7.5 × 10^3^) were plated on the coverslips in media with 10% FBS; incubated for 2, 5 or 24 hours; fixed with 3% formaldehyde; quenched with 0.15 M glycine; permeabilized with 0.2% Triton-X 100; blocked with 5% goat serum; and incubated with mouse anti-cortactin (1:200, clone 4F11; EMD Millipore, Billerica, MA, USA), mouse anti-vinculin (1:500, clone V284; EMD Millipore) and rabbit anti-paxillin pY118 (1:200, polyclonal; BioSource International, Camarillo, CA, USA), followed by washing and incubating with either goat anti-mouse rhodamine red or donkey anti-rabbit tetramethylrhodamine (1:250). Coverslips were mounted and viewed at 400× magnification with an inverted fluorescence microscope (Nikon Eclipse TE300; Nikon Instruments, Melville, NY, USA). Images were digitally acquired and processed with MetaMorph software (Molecular Devices, Sunnyvale, CA, USA) and ImageJ software (National Institutes of Health, Bethesda, MD, USA).

### Focal adhesion kinase depletion *in vivo*

To delete fak, mice were injected intraperitoneally at 6 weeks of age (when arthritis is typically not clinically apparent) with 1 mg of tamoxifen in 1 ml of sunflower oil a total of three times with each dose 3 days apart. All experiments were performed using TNF^+^CreER^+^FAK^f/f^ mice treated with tamoxifen and littermate controls that were either TNF^+^CreER^+^FAK^f/f^ mice treated with sunflower oil or TNF^+^CreER^−^FAK^f/f^ mice treated with tamoxifen. No difference was seen using these two different controls, and data were pooled.

### Clinical arthritis scores

Arthritis was scored by the same investigator in a blinded manner on a scale of 0 to 3 as described previously [34], with 0 = no arthritis; 0.5 = mild joint deformity, mild swelling; 1.0 = moderate joint deformity, moderate swelling; 1.5 = moderate/severe joint deformity, moderate swelling, decreased grip strength on a metal wire; 2.0 = severe joint deformity, moderate swelling, no grip strength; 2.5 = severe joint deformity, moderate/severe swelling, no grip strength, and 3.0 = severe joint deformity and swelling, no grip strength.

### Pathology

Hind legs were fixed in neutral buffered 10% formalin and decalcified with Surgipath Decalcifier I (Leica Biosystems, Buffalo Grove, IL, USA) for 30 hours. Tissue was embedded, sectioned and stained with hematoxylin and eosin (H&E) using standard methods. The tibiotalar joint was scored by a single pathologist who was blinded to the genotype of the mice. The pathology scoring scale for synovitis is as follows: 0 = none, 1 = mild, 2 = moderate, 3 = moderately severe and 4 = severe. The severity scale for erosion is as follows: 0 = none, 1 = erosion of one surface of the tibiotalar joint with less than 50% involvement, 2 = erosion of less than 50% of both surfaces of the tibiotalar joint, 3 = erosion of more than 50% of both surfaces of the tibiotalar joint and 4 = complete destruction of the tibiotalar joint.

### Statistics

Significance was determined using one-way analysis of variance with *post hoc* tests or paired and unpaired *t*-tests as appropriate (GraphPad Prism software; GraphPad Software, La Jolla, CA, USA).

## Results

**Figure 1:**
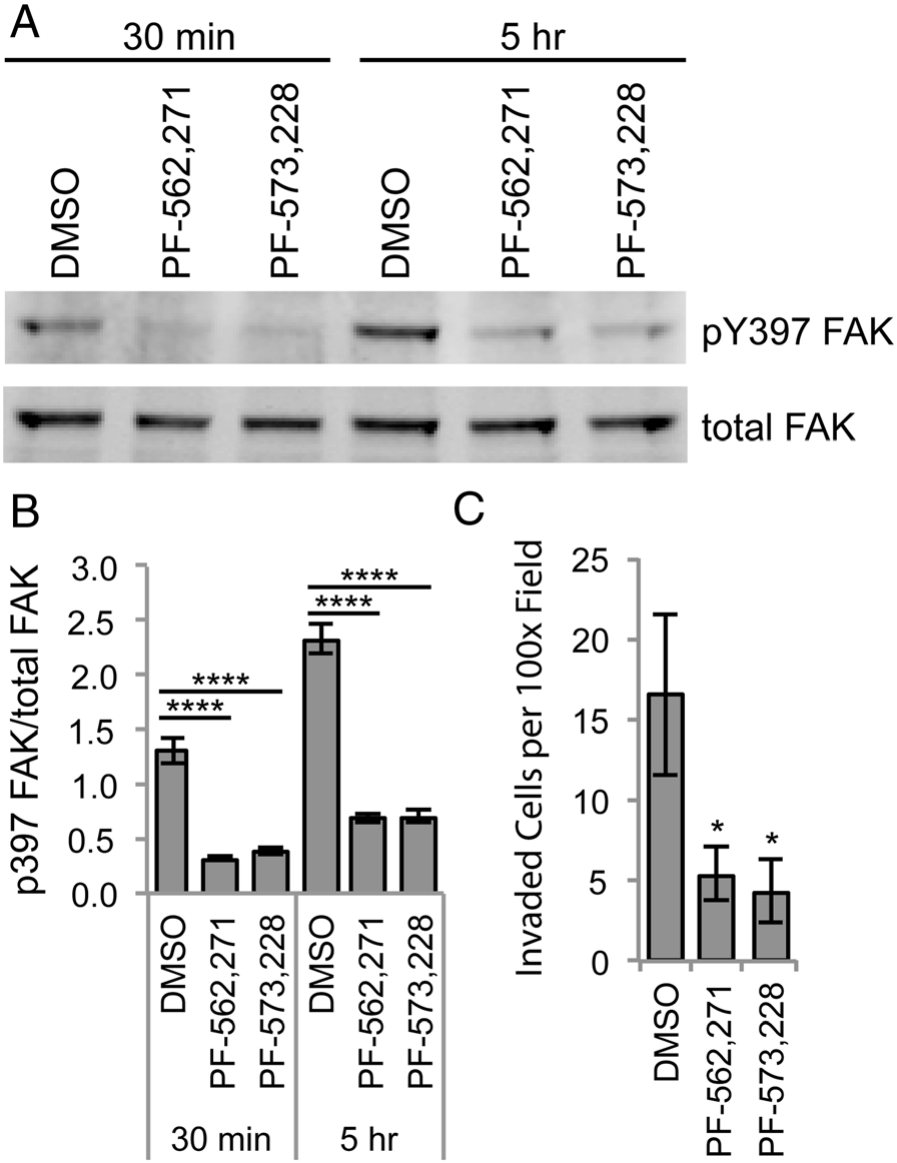
**Focal adhesion kinase inhibitors reduce synovial fibroblast invasion** ***in vitro*** **.** Human rheumatoid synovial fibroblasts were treated with focal adhesion kinase (FAK) inhibitors for 30 minutes or 5 hours. Cells were lysed and subjected to Western blot analysis for total FAK and FAK phosphorylated at tyrosine 397 (pY397). Bands were quantified by densitometry. Representative blot is shown in **(A)**. **(B)** Graph depicts phosphorylated FAK divided by total FAK (*n* = 3 independent experiments using cell lines from two different patients). **(C)** Rheumatoid synovial fibroblasts were treated with PF-562,271, PF-573,228 or dimethyl sulfoxide (DMSO) as the vehicle control and allowed to invade for 24 hours in Matrigel invasion chambers. Graph shows the number of invaded cells per microscopic field at 100× magnification (*n* = 4 replicates using cell lines from three different patients). All graphs show average ± standard error of the mean (SEM) data with *P < 0.05 and ****P < 0.0001 by one-way analysis of variance.

To assess whether FAK activation is important for synovial fibroblast invasion, we first confirmed inhibition of FAK in rheumatoid synovial fibroblasts. Synovial fibroblasts derived from the synovial fluid of patients with rheumatoid arthritis were treated for either 30 minutes or 5 hours with vehicle control or either of the FAK inhibitors PF-562,271 or PF-573,228. Cell lysates were subjected to Western blot analysis to assess levels of FAK phosphorylated at tyrosine 397. As shown in Figures 1A and 1B, at both 30 minutes and 5 hours, there was a significant reduction in pY397 FAK with FAK inhibition compared to vehicle control. Next, synovial fibroblasts were treated with DMSO, PF-562,271 or PF-573,228 and allowed to invade for 24 hours through Matrigel invasion chambers. As shown in Figure 1C, there was a threefold reduction in the ability of FAK-inhibited synovial fibroblasts to invade. Thus, activated FAK appears to be important for the invasive capability of human rheumatoid synovial fibroblasts through a three-dimensional matrix *in vitro*.

**Figure 2:**
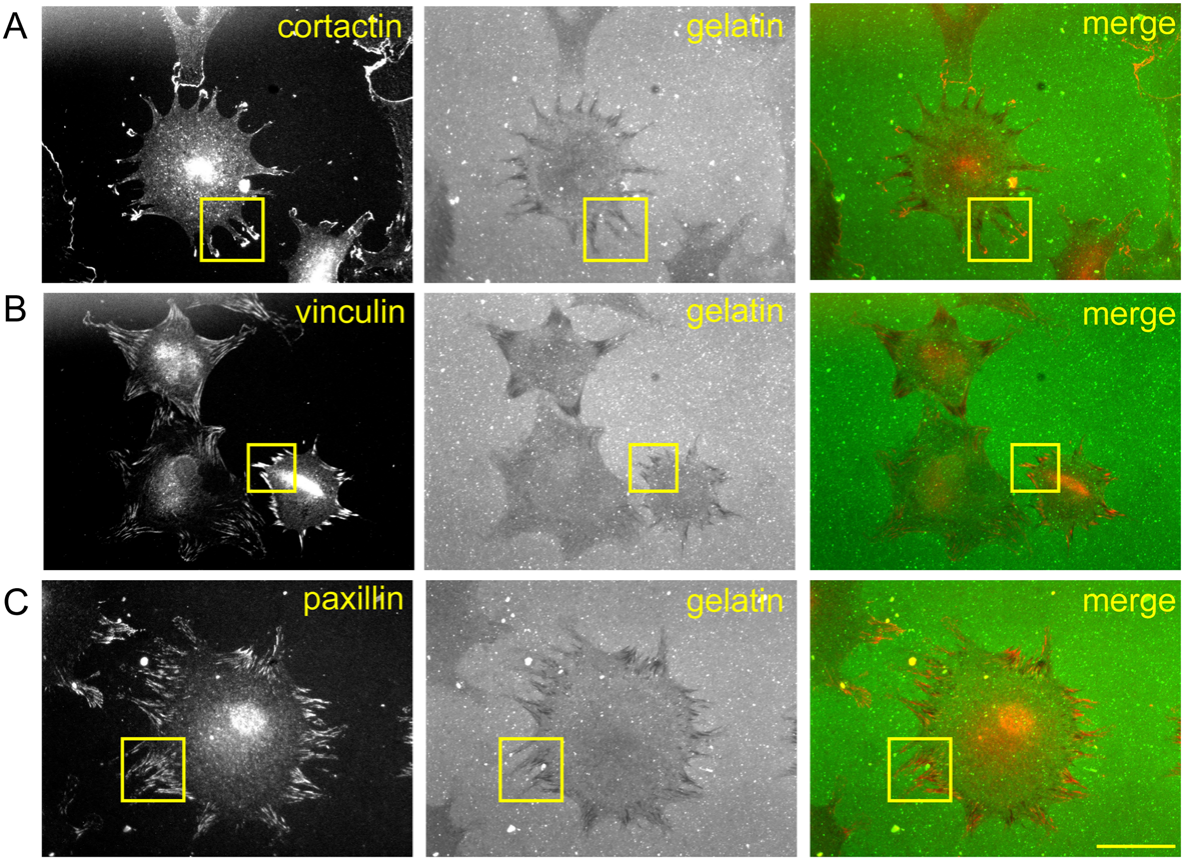
**Rheumatoid synovial fibroblasts degrade centrally and at focal adhesions.** Rheumatoid synovial fibroblasts were allowed to adhere for 2 hours to coverslips coated with fluorescently labeled gelatin. Coverslips were fixed and stained for cortactin, vinculin and paxillin. Images are representative of gelatin degradation and staining for cortactin **(A)**, vinculin **(B)** and paxillin **(C)** in three independent experiments in which we used rheumatoid synovial fibroblasts from two different patients. Images were made at 400× magnification, and the bar represents 50 μm. To demonstrate degradation at focal adhesions, boxes indicate examples of areas where gelatin degradation *did not* colocalize with cortactin **(A)**, a marker of invadopodia, as well as where gelatin degradation *did* colocalize with vinculin **(B)** and paxillin **(C)**, two markers of focal adhesions.

Two major components of cellular invasion are degradation of extracellular matrix and migration. Given the deficits seen in invasion after FAK inhibition, we sought to determine whether these components of invasion would be affected by FAK inhibitors. We first addressed matrix degradation and started by characterizing the pattern of degradation in rheumatoid synovial fibroblasts. We plated rheumatoid synovial fibroblasts on fluorescent gelatin-coated coverslips and allowed them to degrade for 2 hours. We then fixed the cells and stained them for cortactin, a cytoskeletal protein found in invadopodia [35]. As shown in Figure 2A, gelatin degradation was seen in the central portion of many cells colocalizing with cortactin staining, consistent with the invadopodia previously reported in synovial fibroblasts [5]. However, we also saw degradation that did not colocalize with cortactin at the cell periphery, where focal adhesions are located. Similar patterns were seen at the 5- and 24-hour time points (data not shown). To determine whether the peripherally degrading structures were focal adhesions, we repeated the degradation experiments and stained for vinculin and paxillin, two cytoskeletal proteins typically seen in focal adhesions [36]. As shown in Figures 2B and 2C, staining for vinculin and paxillin aligned with the peripheral degradation areas, suggesting that rheumatoid synovial fibroblasts may use both invadopodia and focal adhesions to degrade matrix, similarly to cancer cells [8].

**Figure 3:**
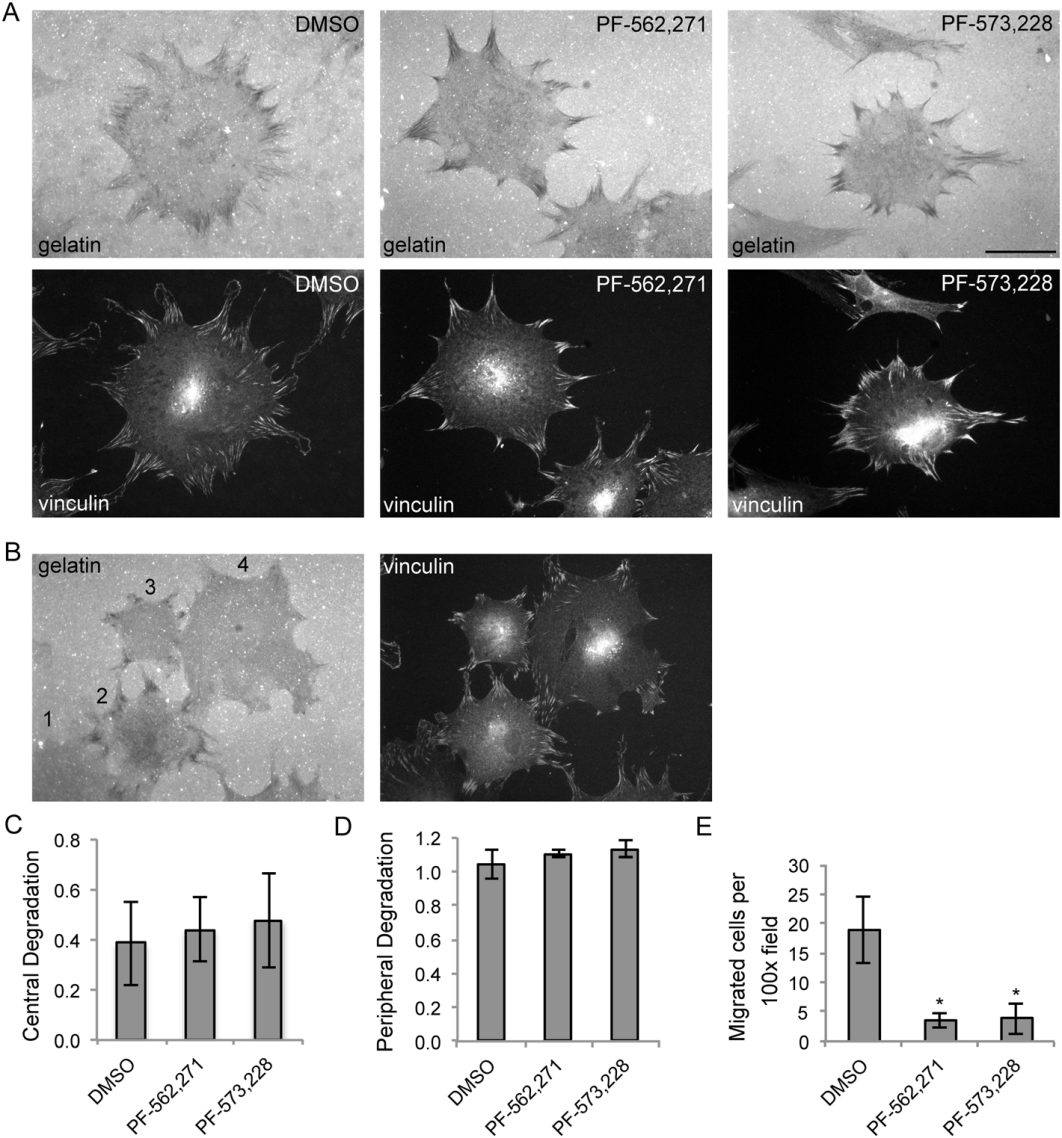
**Focal adhesion kinase inhibitors do not alter focal matrix degradation, but do reduce migration of rheumatoid synovial fibroblasts. (A)** Rheumatoid synovial fibroblasts were treated with PF-562,271, PF-573,228 or dimethyl sulfoxide (DMSO) as the vehicle control, plated on fluorescently labeled gelatin, fixed after 5 hours and stained for vinculin. Representative images of matrix-degrading cells from each condition are displayed (*n* = 3 independent experiments using cell lines from two different patients). Images were created at 400× magnification, and the bar represents 50 μm. **(B)** Left panel depicts a scoring system for gelatin degradation: *1* = no peripheral degradation (*note*: for quantitative analyses in **(C)** and **(D)**, only cells that were completely within the field of view were scored), *2* = central and severe peripheral degradation, *3* = severe peripheral degradation and no central degradation and *4* = mild peripheral degradation and no central degradation. Cells treated as in **(A)** were scored for degradation as described in for **(B)**. **(C)** and **(D)** Graphs show the average scores ± SEM for central **(C)** and peripheral **(D)** degradation (*n* = 3 independent experiments using cell lines from two different patients). **(E)** Rheumatoid synovial fibroblasts were treated with PF-562,271, PF-573,228 or vehicle control (DMSO) and allowed to migrate for 5 hours across a transwell. The graph shows average number ± SEM of migrated cells per microscopic field at 100× magnification (*n* = 4 independent experiments using cell lines from three different patients, *P < 0.05 by one-way analysis of variance).

Having characterized the degradative structures of these rheumatoid arthritis synovial fluid–derived synovial fibroblasts, we next sought to determine whether FAK inhibitors could alter focal matrix degradation. Rheumatoid synovial fibroblasts were treated with FAK inhibitors or vehicle control and allowed to degrade fluorescent gelatin for 5 hours. Coverslips were stained for vinculin to localize the structures of the cells. As shown in Figure 3A, FAK-inhibited cells appear to have normal matrix degradation. We then scored the cells for degradation as outlined in Figure 3B. Central degradation appeared to either be present or absent, so synovial fibroblasts were scored with either a 0 or a 1 for the absence or presence of degradation, respectively. As shown in Figure 3C, there was no difference in central degradation scores upon FAK inhibition. There was more variation in the extent of degradation at the periphery, so this was scored as 0 (none), 1 (mild) or 2 (severe) for each cell. There was no difference in degradation at focal adhesions with FAK inhibition (Figure 3D). Similar results were seen after 24 hours of incubation of synovial fibroblasts on gelatin (data not shown).

We then sought to determine whether FAK inhibition could alter rheumatoid synovial fibroblast migration. Rheumatoid synovial fibroblasts were treated with FAK inhibitors or vehicle control and allowed to migrate across fibronectin-coated transwells for 5 hours. As shown in Figure 3E, migration was impaired when FAK was inhibited. Taken together, these data suggest that FAK inhibitors impair the migratory component of synovial fibroblast invasion, but not focal matrix degradation.

Because FAK inhibitors reduced rheumatoid synovial fibroblast invasion and migration, which are two important components of rheumatoid arthritis progression, we evaluated whether FAK protein itself contributes to synovial fibroblast invasion and arthritis severity. To do so, we generated a mouse model of arthritis in which fak could be deleted upon exposure to tamoxifen. We crossed TNFα-overexpressing mice, which develop a chronic inflammatory, erosive arthritis similar to rheumatoid arthritis [29], with FAK^f/f^ mice, in which the *fak* gene is floxed [30], and Rosa26-CreER^T2^ mice [31], in which Cre recombinase deletes floxed genes upon exposure to tamoxifen. We ultimately generated TNF^+^CreER^+^FAK^f/f^ and TNF^+^CreER^−^FAK^f/f^ mice for experiments.

**Figure 4:**
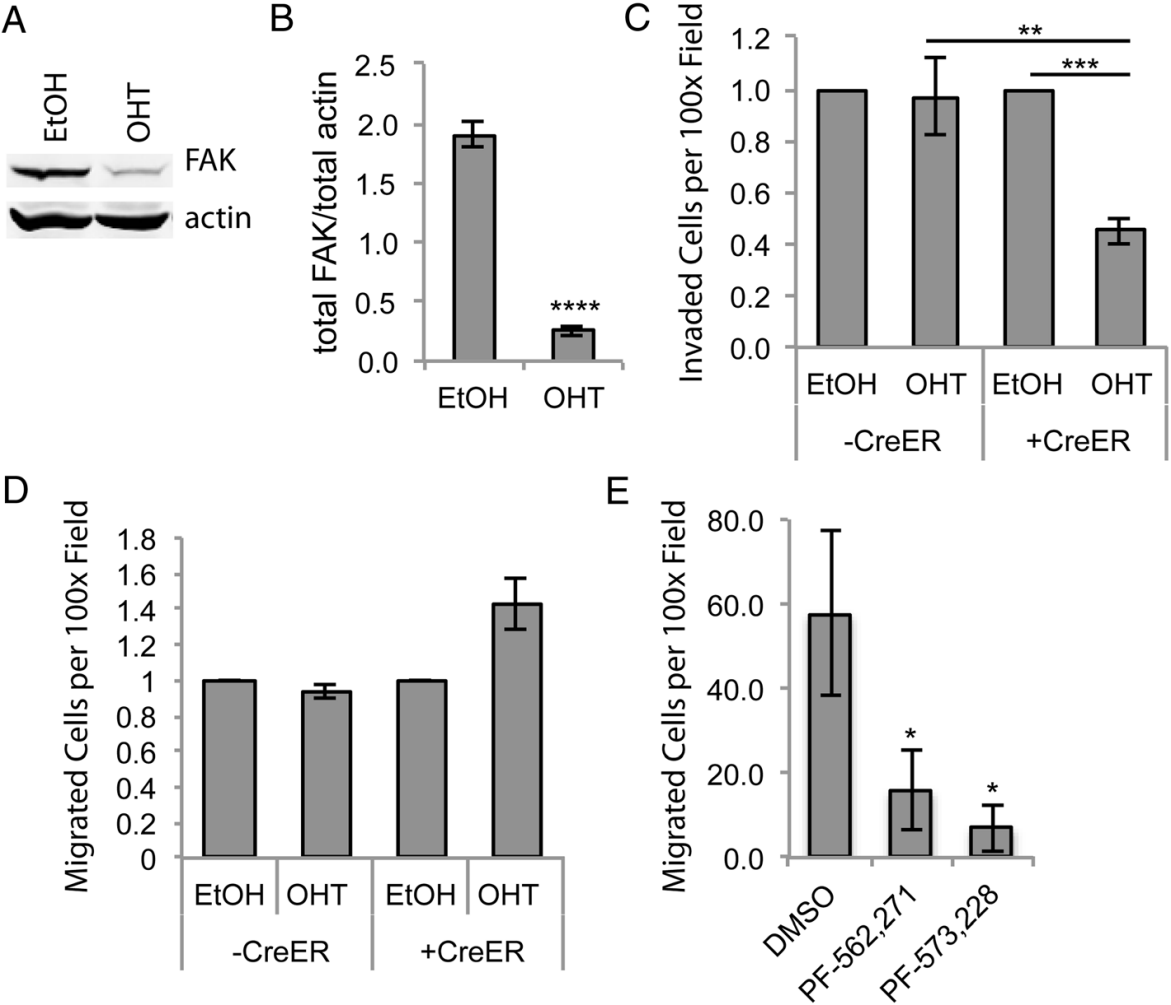
**Focal adhesion kinase depletion reduces murine arthritic synovial fibroblast invasion, but not migration,** ***in vitro*** **.** TNF^+^CreER^+^FAK^f/f^ murine synovial fibroblasts were treated with either (Z)-4-OH tamoxifen (OHT) or 95% ethanol vehicle control (EtOH). Cell lysates were subjected to Western blot analysis for focal adhesion kinase (FAK) and actin to test for FAK depletion. **(A)** A representative blot from seven experiments is presented. **(B)** Densitometry was performed, and the average ± SEM for data for the amount of FAK divided by actin are shown (*n* = 7, ****P < 0.0001 by *t*-test). **(C)** TNF^+^CreER^+^FAK^f/f^ (+CreER) and TNF^+^CreER^−^FAK^f/f^ or TNF^+^CreER^−^FAK^+/+^ (−CreER) murine synovial fibroblasts were treated with OHT or EtOH for 4 days and then allowed to invade for 24 hours through Matrigel invasion chambers. The number of invaded cells per microscopic field at 100× magnification was counted, and OHT-treated samples were normalized to EtOH-treated controls. Graph depicts average ± SEM (*n* = 3, **P < 0.01, ***P < 0.005 by one-way analysis of variance (ANOVA)). **(D)** +CreER and –CreER murine synovial fibroblasts were treated as in **(C)** and allowed to migrate across fibronectin-coated transwells for 4 hours. The number of migrated cells per microscopic field at 100× magnification was counted, and OHT-treated samples were normalized to EtOH-treated controls. Graph depicts average ± SEM data (*n* = 3). **(E)** Murine TNFα-overexpressing synovial fibroblasts were treated with dimethyl sulfoxide (DMSO) vehicle control, PF-562,271 or PF-573,228 and allowed to migrate across fibronectin-coated transwells for 4 hours. The number of invaded cells per microscopic field at 100× magnification was counted. Graph depicts average ± SEM data (*n* = 4, *P < 0.05 by one-way ANOVA).

Before addressing arthritis, we evaluated whether FAK protein is important for murine arthritic synovial fibroblast invasion. We generated synovial fibroblasts from TNF^+^CreER^+^FAK^f/f^ and TNFα-overexpressing mice that lacked Cre recombinase. TNF^+^CreER^+^FAK^f/f^ synovial fibroblast cultures were treated *in vitro* with (Z)-4-OH tamoxifen or vehicle control for 4 days and cell lysates were analyzed by western blot to quantify the loss of FAK. As shown in Figure 4A and B, there was a dramatic reduction in FAK protein in the TNF^+^CreER^+^FAK^f/f^ synovial fibroblasts treated with (Z)-4-OH tamoxifen. We then treated TNF^+^CreER^+^FAK^f/f^ and TNFα-overexpressing synovial fibroblasts without Cre with either (Z)-4-OH tamoxifen or vehicle control for 4 days, followed by subjecting the synovial fibroblasts to the Matrigel invasion chamber assay as described above. As shown in Figure 4C, invasion was impaired only in the TNF^+^CreER^+^FAK^f/f^ synovial fibroblasts treated with (Z)-4-OH tamoxifen, suggesting that FAK is necessary for efficient murine synovial fibroblast invasion into three-dimensional matrix *in vitro*.

We also tested whether FAK protein would be required for murine arthritic synovial fibroblast migration. TNF^+^CreER^+^FAK^f/f^ and TNFα-overexpressing synovial fibroblasts without Cre were treated with either (Z)-4-OH tamoxifen or vehicle control as described above and then allowed to migrate across fibronectin-coated transwells for 4 hours. Interestingly, in contrast to the reduction in migration in the presence of FAK inhibitors, there was no defect in murine synovial fibroblast migration after deletion of fak (Figure 4D). To be sure that this discrepancy was not related to the difference in species, type of arthritis or source of synovial fibroblasts (synovial fluid versus synovial tissue), we treated murine arthritic synovial fibroblasts with DMSO, PF-562,271 or PF-573,228 (as described above) and then allowed them to migrate across fibronectin-coated transwells for 4 hours. As shown in Figure 4E, synovial fibroblast migration was reduced with the FAK inhibitors. Taken together, these data suggest that although FAK inhibitors reduce synovial fibroblast invasion and migration, FAK itself is required for synovial fibroblast invasion into three-dimensional matrix, but is not required for two-dimensional migration on fibronectin.

**Figure 5:**
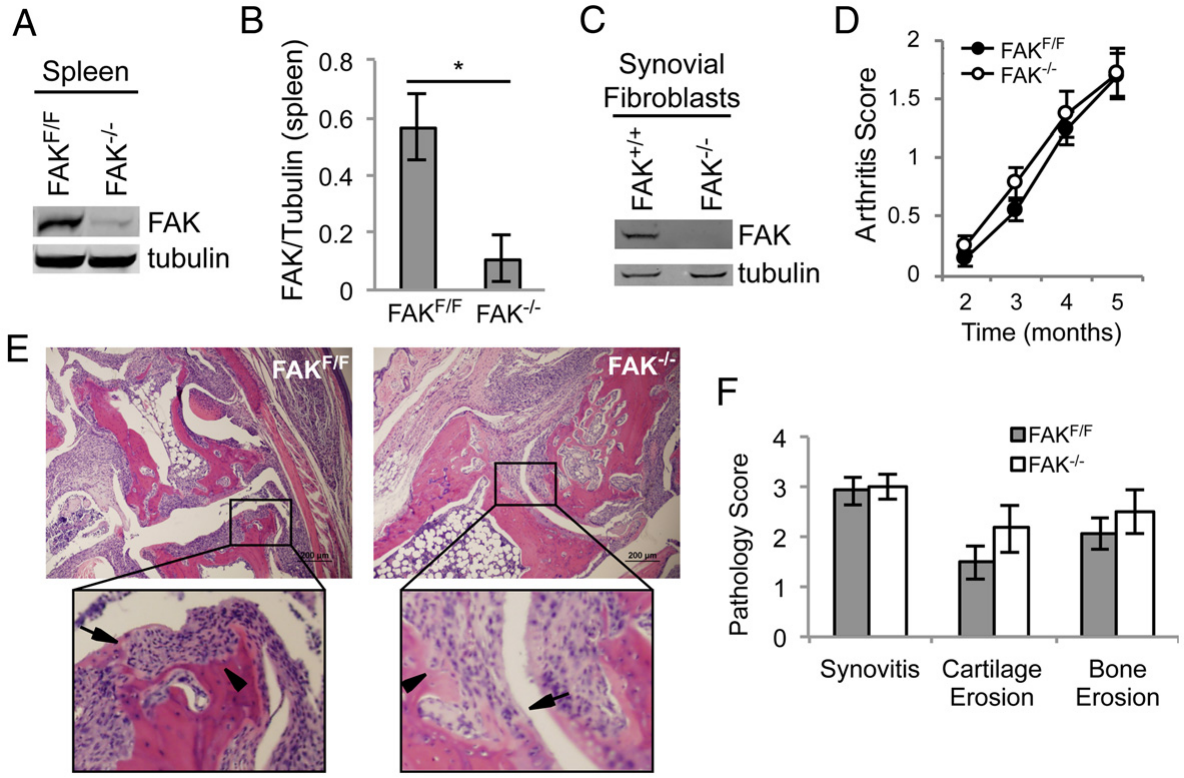
**Focal adhesion kinase depletion does not reduce synovial fibroblast invasion into cartilage and bone or clinical arthritis in murine tumor necrosis factor α–induced arthritis.** TNF^+^CreER^+^FAK^f/f^ mice were treated with tamoxifen at 6 weeks of age (FAK^−/−^). For each experimental mouse, there was a littermate control (FAK^f/f^) that was either a TNF^+^CreER^+^FAK^f/f^ mouse treated with vehicle or a TNF^+^CreER^−^FAK^f/f^ mouse treated with tamoxifen. Spleen lysates from FAK^f/f^ and FAK^−/−^ mice at 5 months of age were subjected to Western blot analysis to detect focal adhesion kinase (FAK) and tubulin. A representative blot is presented in **(A)**. **(B)** Densitometry was performed, and the average ± SEM data for FAK levels normalized to tubulin are presented in the graph (*n* = 3, *P < 0.05 by *t*-test). **(C)** Synovial fibroblasts derived from the tibiotalar joints of a 5-month-old FAK^−/−^ mouse and a FAK^+/+^ mouse were expanded to passage 3. Lysates from these synovial fibroblasts were subjected to Western blot analysis for FAK and tubulin. **(D)** Arthritis was clinically scored in FAK^−/−^ and littermate control FAK^f/f^ mice based on hind foot deformity, swelling and grip strength on a scale of 0 to 3. Average ± SEM data are presented in the graph (*n* = 12 sex-matched pairs at 2, 3 and 4 months and *n* = 8 pairs at 5 months). The tibiotalar joint was fixed, decalcified, embedded and sectioned for hematoxylin and eosin staining when the mice were 5 months of age. Representative images are shown in **(E)**. Inset shows areas of pannus invading cartilage (arrows) and bone (arrowheads). Bar represents 200 μm. **(F)** Joints were scored for severity of synovitis, cartilage erosion and bone erosion on a scale of 0 to 4, with average ± SEM data presented in the graph (*n* = 6 pairs).

Because murine arthritic synovial fibroblasts had impaired invasion upon FAK depletion, we sought to determine if TNFα-induced arthritis would be lessened in the absence of FAK. TNF^+^CreER^+^FAK^f/f^ mice were treated with tamoxifen at 6 weeks of age. For controls, TNF^+^CreER^+^FAK^f/f^ littermates were treated with vehicle alone and TNF^+^CreER^−^FAK^f/f^ littermates were treated with tamoxifen. To confirm depletion of FAK, spleens were harvested and subjected to Western blot analysis. As shown in Figures 5A and 5B, there was a significant reduction in FAK levels upon tamoxifen treatment. We attempted to detect depletion of FAK in the joint, but FAK levels in arthritic mice not treated with tamoxifen were below the level of detection of our Western blots. Therefore, we generated a line of synovial fibroblasts from a 5-month-old CreER^+^FAK^f/f^ mouse treated with tamoxifen as described above. At passage 3, lysates were made and subjected to Western blot analysis. As shown in Figure 5C, these synovial fibroblasts also showed substantial depletion of FAK protein compared to FAK levels in normal synovial fibroblasts. Similar results were seen at earlier time points after tamoxifen treatment (data not shown).

Having confirmed FAK depletion, we clinically scored arthritis monthly until the mice were 5 months of age. The scoring was done in a blinded manner at the hind feet based on joint deformity, swelling and grip strength. As shown in Figure 5D, there was no significant difference in clinical arthritis in the absence of FAK. When the mice were 5 months of age, their hind legs were fixed, decalcified, sectioned and stained with H&E (Figure 5E). The tibiotalar joints were scored in a blinded manner based on synovitis and joint erosion. As shown in Figure 5F, there was no difference in either synovitis or erosive disease in the absence of FAK. Thus, although FAK is required *in vitro* for synovial fibroblast invasion, it is dispensable for inflammatory arthritis and joint erosions.

## Discussion

In this study, we have demonstrated that FAK is required for arthritic synovial fibroblast invasion *in vitro* (Figures 1 and 4). FAK has already been shown to be important for cytokine production in synovial fibroblasts [19,20], but, to our knowledge, this study is the first to show FAK’s role in invasion in these cells. However, despite the significant reduction in synovial fibroblast invasion *in vitro* when FAK is inhibited or depleted and in spite of the known role for synovial fibroblasts in erosive disease [2,3], joint erosions in TNFα-induced inflammatory arthritis were unaltered after deletion of FAK (Figure 5), suggesting different molecular requirements for cellular invasion *in vitro* versus *in vivo*.

There are several possible reasons why inflammatory erosive arthritis is not altered in FAK-deficient mice. First, although we observed that depletion of FAK was quite good, there may still have been some residual FAK allowing disease progression. Second, it is possible that this model of arthritis is not FAK-dependent. Third, *in vitro* invasion was reduced, not eliminated, in the absence of FAK, and the residual amount of invasion may have been sufficient for arthritis and joint erosions. Fourth, we found that activated FAK was not required for focal matrix degradation in rheumatoid synovial fibroblasts (Figure 3). Similar phenomena were seen in macrophages in which FAK was required for invasion, but not focal matrix degradation by podosomes [22]. In breast cancer cells, FAK is required for invasion, but is a negative regulator of invadopodia formation [37]. When FAK is depleted in human fibrosarcoma cells, more than half of cells can still degrade at focal adhesions [8]. Thus, perhaps, joint invasion depends primarily on matrix degradation, which is FAK-independent. In locations where the synovium contacts the joint, invasion occurs slowly over weeks and months. Perhaps bone and cartilage can be destroyed simply by local, constant degradation.

A final possible reason why murine inflammatory arthritis may not be reduced upon depletion of FAK may be that Pyk2 (proline-rich tyrosine kinase), a homologue of FAK, can compensate for loss of FAK. Rheumatoid synovial tissue has elevated phosphorylated Pyk2, and TNFα can induce Pyk2 phosphorylation in rheumatoid synovial fibroblasts [17]. Further, like FAK, Pyk2 is important for T-cell, neutrophil and macrophage functions [38-40]. In a previous study, when FAK-related nonkinase (FRNK), an endogenous FAK inhibitor, was overexpressed intraarticularly in a model of rat arthritis, bone erosions and osteoclasts were reduced [25]. However, FRNK has been shown to inhibit both FAK and Pyk2 [41], and FAK and Pyk2 function synergistically in osteoclast podosome formation [42]. Therefore, FAK and Pyk2 may have overlapping roles in inflammatory arthritis. We have looked at Pyk2 levels in synovial fibroblasts in which FAK has been deleted and have seen no changes in Pyk2 levels (data not shown). However, even without a change in Pyk2 levels, the normal amount of Pyk2 might be sufficient to compensate for loss of FAK in inflammatory arthritis.

We also found that FAK inhibitors reduced synovial fibroblast migration, but that FAK protein itself did not appear to be required. This is surprising because FAK is required for migration in other cell types, such as macrophages, although in the macrophage experiments described in [22], transwells were not coated with fibronectin, which might lead to an altered need for FAK. It is possible that residual amounts of FAK after deletion were sufficient to allow migration. However, FAK depletion appeared more robust (Figure 4) than FAK inhibition (Figure 1). Another possibility is that cells may compensate for the loss of FAK better than inhibition of FAK, because, in the case of inhibition, the FAK molecule is still present and cannot necessarily be replaced by a similar protein. One final explanation for the discrepancy between the FAK inhibitors and FAK depletion is that FAK inhibitors also inhibit Pyk2 and several cyclin-dependent kinases [43]. Perhaps inhibition of at least one of these other molecules is necessary in addition to inhibition of FAK itself to reduce synovial fibroblast migration in transwell experiments. Such off-target effects of the FAK inhibitors may contribute to their efficacy in cancer and may also make them efficacious in rheumatoid arthritis, despite the fact that depletion of FAK alone appears to have no effect in synovial fibroblast migration or arthritis.

We have further characterized the structures used by rheumatoid synovial fibroblasts to invade. Others have reported that arthritic synovial fibroblasts make invadopodia [5,6]. We did see central areas of degradation colocalizing with cortactin, but we also saw degradation at focal adhesions. Because degradation at focal adhesions has not been reported previously in human synovial fibroblasts, it is possible that this is a unique feature of synovial fibroblasts isolated from synovial fluid. However, this is unlikely because peripheral degradation also occurs in murine arthritic synovial fibroblasts generated from synovial tissue [33]. To the best of our knowledge, we are the first to observe focal adhesion degradation in nonmalignant human cells, which is interesting, given the comparisons made between the rheumatoid pannus and tumors [1]. Therefore, degradation at focal adhesions may be a special pathologic feature of rheumatoid synovial fibroblasts.

## Conclusions

We show that FAK contributes to synovial fibroblast invasion, but is dispensable in TNFα-driven erosive arthritis. Further, we found that rheumatoid synovial fibroblasts appear to degrade at both invadopodia and focal adhesions, which may contribute to the pathophysiology of rheumatoid arthritis. Further studies are needed to address the role of Pyk2 and other mediators of cellular invasion and migration in inflammatory arthritis.

## Abbreviations

ANOVA: Analysis of variance
DMSO: Dimethyl sulfoxide
FAK: Focal adhesion kinase
FBS: Fetal bovine serum
FRNK: Focal adhesion kinase–related nonkinase
H&E: Hematoxylin and eosin
PAMP: Pathogen-associated molecular pattern
PBS: Phosphate-buffered saline
Pyk2: Proline-rich tyrosine kinase
SEM: Standard error of the mean
TNFα: Tumor necrosis factor α
